# Final-year information on didactic and organizational issues for students and supervising physicians – project report on the development and implementation of the cross-site website PJ-input

**DOI:** 10.3205/zma001588

**Published:** 2023-02-15

**Authors:** Angelika Homberg, Elisabeth Narciß, Julia Thiesbonenkamp-Maag, Felix Heindl, Katrin Schüttpelz-Brauns

**Affiliations:** 1 Medical Faculty Mannheim, Heidelberg University, Division for Study and Teaching Development, Department of Medical Education Research, Mannheim, Germany; 2 Medical Faculty Mannheim, Heidelberg University, Competence Center for final-year education Baden-Württemberg, Mannheim, Germany; 3 Medical Faculty Mannheim, Heidelberg University, Division for Study and Teaching Development, Mannheim, Germany; 4 Medical Faculty Ulm, Competence Center eEducation Baden-Württemberg, Ulm, Germany

**Keywords:** final year, internet, undergraduate medical education, access to information, preceptorship, clinical competence

## Abstract

**Objective:**

Final-year training is becoming increasingly important in medical studies and requires a high degree of personal responsibility from students. It is the task of supervising physicians to make informal learning opportunities available to students when working with and on patients and to gradually transfer responsibility to them. Both students and physicians have a great need for information regarding the contextual conditions and didactic realization of this transfer of responsibility. Up to now, the faculties have only provided information and support in a sporadic manner and with little standardization. With MERLIN, the joint project undertaken by the Competence Network for Teaching Medicine in Baden-Württemberg, a platform for the final year was developed and released on the web. The aim was to bundle information in order to support students and supervising physicians in their teaching-learning process and to improve the quality of teaching in the final year.

**Project description:**

The development process of this platform took place in several steps across all faculties. Content and materials were compiled and structured based on a needs assessment. The first draft was evaluated by means of a simulation by students and then revised. A professional internet agency was involved for the technical implementation. The newly designed website *PJ-input* (“PJ” being the abbreviation for “Praktisches Jahr”, the final year) contains areas for students and supervising physicians, as well as faculty-specific and general information about the final year. Faculty-specific content can be entered directly by the respective staff via an input mask and updated at any time. The provision of didactic materials can support competency-oriented teaching and learning in the final year. Here, for example, the concept of the Entrustable Professional Activities (EPA) was taken up, which gives students and supervising physicians orientation for the gradual assumption or transfer of responsibility. The platform was launched in spring 2021. Usage behavior is continuously recorded via the web application.

**Results and conclusion:**

The evaluation results show that the website is visited often and perceived as supportive. Increasing usage figures and the high frequency of use by students in the sections “im PJ” (during the final year) and “nach dem PJ” (after the final year) for the faculties involved in the MERLIN project confirm the target group-oriented design and use. The site should be promoted even more to pre-final-year students, as well as across state borders and to the target group of faculties. It is expected that nationwide faculty participation will make a significant contribution to the competency-based shift in teaching and the standardization of training during the final year of study under the new licensing regulations.

## Zusammenfassung

**Zielsetzung:**

Die Ausbildung im Praktischen Jahr (PJ) gewinnt im Medizinstudium an Bedeutung und verlangt von Studierenden ein hohes Maß an Eigenverantwortung. Ausbildende Ärztinnen und Ärzte haben die Aufgabe, informelle Lernmöglichkeiten bei der Arbeit mit und an Patientinnen und Patienten für Studierende nutzbar zu machen und ihnen schrittweise Verantwortung zu übertragen. Sowohl Studierende als auch Ärztinnen und Ärzte haben einen hohen Informationsbedarf in Bezug auf Rahmenbedingungen oder zur didaktischen Umsetzung der Verantwortungsübergabe. Entsprechende Informationen und Unterstützungsangebote wurden bislang seitens der Fakultäten nur punktuell und wenig standardisiert bereitgestellt. Im Verbundprojekt MERLIN des „Kompetenznetzes Lehre in der Medizin Baden-Württemberg“ wurde eine Plattform für das PJ entwickelt und im Web freigeschaltet. Ziel war die Bündelung von Informationen, um Studierende und Lehrende in ihrem Lehr-Lernprozess zu unterstützen und die Qualität der Lehre im PJ zu verbessern.

**Projektbeschreibung:**

Der Entwicklungsprozess dieser Plattform verlief standortübergreifend in mehreren Schritten. Ausgehend von einer Bedarfsanalyse wurden Inhalte und Materialien zusammengestellt und strukturiert. Dieser Entwurf wurde mittels einer Simulation durch Studierende evaluiert und anschließend überarbeitet. Für die technische Umsetzung wurde eine professionelle Internetagentur eingebunden. Die neu konzipierte Website *PJ-input* enthält Bereiche für Studierende und ausbildende Ärztinnen und Ärzte sowie fakultätsspezifische und übergreifende Informationen rund um das PJ. Die fakultätsspezifischen Inhalte können hierbei über eine Eingabemaske direkt von den jeweiligen Standorten eingegeben und jederzeit aktualisiert werden. Durch die Bereitstellung didaktischer Materialien kann das kompetenzorientierte Lehren und Lernen im PJ unterstützt werden. Hierbei wurde beispielsweise das Konzept der Anvertraubaren Professionellen Tätigkeiten (APT) aufgegriffen, welches Studierenden und Lehrenden Orientierung für die schrittweise Übernahme bzw. Übergabe von Verantwortung gibt. Die Plattform wurde im Frühjahr 2021 freigeschaltet. Das Nutzungsverhalten wird kontinuierlich über die Webanwendung erfasst.

**Ergebnisse und Schlussfolgerung:**

Die Evaluationsergebnisse zeigen, dass die Website gut besucht und als unterstützend wahrgenommen wird. Steigende Nutzungszahlen und die starke Frequentierung durch Studierende in den Bereichen „im PJ“ und „nach dem PJ“ der im MERLIN-Projekt beteiligten Fakultäten bestätigen die zielgruppenorientierte Ausgestaltung und Nutzung. Die Seite sollte noch stärker für Studierende vor dem PJ beworben werden, sowie über die Landesgrenze hinweg und bei der Zielgruppe der Lehrenden. Es wird erwartet, dass eine deutschlandweite Einbindung der Fakultäten vor dem Hintergrund der neuen Approbationsordnung zur kompetenzorientierten Umgestaltung der Lehre und Standardisierung der Ausbildung im PJ einen wesentlichen Beitrag leistet.

## Introduction

Various social and political developments influence healthcare provision and also have an indirect impact on training in the healthcare professions. For example, new licensing regulations for dentists and for psychotherapists were passed on September 22, 2021. The final year of training as the last stage of medical studies is also subject to continuous processes of change and adaptation, for example, due to the nationwide mobility in the final year introduced by the 2013 amendment (of July 17, 2012) to the 2002 licensing regulations for physicians (Approbationsordnung für Ärztinnen und Ärzte, ÄApprO) and the obligation to train according to the logbook. Already years ago, calls were made for the development of transferable and substantial learning objectives, as well as a better structuring and standardization of the final year [1], [2]. In 2018 a commission of experts for the 2020 Master Plan for Medical Studies has drawn up recommendations for restructuring medical studies. These provide for a nationwide restructuring of the final year. For example, a quarter for ambulatory medicine is to be implemented in the future and the goal of independent patient care by final-year students is to be increasingly pursued [3]. While in the current medical licensing regulations two paragraphs provide for the regulation of the final year (ÄApprO 2002, last amended by Art. 3 G v. 16.3.2020 I 497, section 1 §§ 3, 4), in the draft bill for the new licensing regulations, 29 paragraphs are dedicated to this study phase (ÄApprO, November 17, 2020, chapter 1 section 3 §§ 43 ff.). Even if this very detailed regulation of the final year is to be criticized, it must be stated that the potential of final-year training is currently not exploited [4]. The teaching-learning setting in the final year is characterized by working directly on patients in university hospitals, teaching hospitals or teaching practices and differs significantly from that of the preceding years. Most of the teaching takes place locally at the respective place of assignment. Learning cannot be planned for easily because the locations, contact persons, medical staff and patients are constantly changing. This requires a high degree of initiative on the part of the students in order for them to benefit from the predominantly informal learning opportunities [5], [6], [7]. Supervising physicians also face the challenge of preparing students for their future careers in addition to managing their own patient care responsibilities. This is particularly true for the final-year study phase, since training also takes place at teaching hospitals and teaching practices that are otherwise not or hardly involved in student teaching. With the planned introduction of a mandatory quarter in an ambulatory setting, the proportion of these institutions will increase even further. The educational mission goes beyond simply teaching knowledge and skills. Rather, it is about supporting students as they grow into the medical role and assume responsibility, and ultimately about fostering appropriate identity development [8], [9]. Both students and supervising physicians face the challenge of adequately informing themselves and selecting appropriate learning environments and strategies. Students need practice opportunities that are appropriate to their skills and provide the right level of challenge [10]. In addition, there is also a need for continuity, relationship building and establishing trust [6]. Last but not least, the specified learning objectives must be kept in mind, and the formal and legal regulations must be met. Currently in Germany, the teaching standards applicable to the final year are implemented in a largely inconsistent manner [11]. Although each faculty is required to provide logbooks with the educational objectives to be achieved, these have been designed very heterogeneously and pursue different goals [12], [13], [14], [15], [16]. In general, previous logbooks focused on isolated activities, such as drawing blood, performing an anamnesis or a specific examination technique, which students were expected to master at the end of the final year. For this, the didactic three-step approach was established: demonstration, multiple supervised performances, routine. In the future, the emphasis will be on students working more and more independently and assuming responsibility for entire sets of activities. The assignment of these activities will be based on the Entrustable Professional Activities (EPA) [17], [18], [19]. These are parts of authentic medical activities that can be evaluated by observation and gradually entrusted to students [20]. Guidelines for the implementation of EPA in the subjects of surgery, internal medicine, and general medicine have been prepared by a working group of the German Medical Faculty Association (MFT) [https://medizinische-fakultaeten.de/themen/studium/neue-konzepte-fuer-die-ausbildung-im-praktischen-jahr/]. Also, a set of EPAs defines the graduate profile of medical studies [https://www.nklm.de] in the new version of the National Competency-based Catalogue of Learning Objectives for Undergraduate Medical Education (NKLM 2.0) published by the MFT on April 27, 2021. According to the draft bill for the new licensing regulations (ÄApprO 2025), supervising physicians will have a mentoring role in the future (draft bill ÄApprO 2020, § 50).

### MERLIN project: final-year information platform

These developments and influences were taken up in MERLIN, the joint project on competence-oriented learning, teaching and assessing in medicine undertaken by the Competence Network for Teaching Medicine in Baden-Württemberg. One focus was placed on the competence-oriented further development of the final year. In the course of further developing this training phase, instruments were created for a better standardization and structuring of the final-year training and for nationwide use beyond the locations in Baden-Württemberg. The aim was to support the transfer of competencies acquired during the final-year training phase to the everyday working life of a physician. At the same time, the contextual conditions which are conducive to learning and favor the implementation of competency-oriented teaching were investigated [21], [22]. Here it became apparent that flexible supports and clear communication of the legal structures and didactic possibilities are essential, especially due to the time-limited resources of the supervising physicians. In the first project phase, a manual, along with other things, was developed for supervising physicians that includes information on the process, organization, rights and obligations, and teaching opportunities regarding the final year [23]. In the second project phase (2017 to 2021), this and other information was made available to supervising physicians and students via a site-wide website. Providing information and learning materials without access restrictions via the Internet can have positive effects on learning behaviors [24] and offers a long-term, low-cost and low-threshold way to reach a broad readership [25]. 

Based on these considerations, the development and implementation of a non-commercial final-year platform was defined as a work package of the MERLIN project. The website should have a high user and practice orientation and provide both final-year students and supervising physicians with comprehensive, low-threshold and site-wide information and learning materials over the entire course of the final year. The website is intended to assist final-year students in planning and organizing the final year and the self-study phases on their own. The general features of the final year, such as nationwide mobility, are to be taken into account. The website should also contribute to the professionalization of the supervising physicians, inform them about current contexts and support them in the competency-oriented implementation of teaching.

The following traces and reflects on the development, implementation and evaluation of the information platform.

## Project description

**Figure 1:**
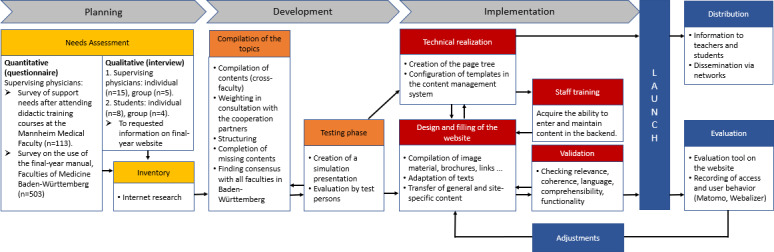
Project steps to develop the information platform for the final year

The planning, development, implementation and evaluation of the information platform was carried out in a four-step process based on already known models of educational needs assessment in the field of higher education didactics and adult education [26], [27], [28]. The individual steps are shown in figure 1 (Fig. 1) and described below.

### Needs assessment

In order to align the website with the target group, information on specific issues was collected, analyzed and reflected upon during the planning phase.

Quantitative and qualitative methods were used to determine the needs. These different methods offer an advantage of both evaluating existing materials, such as the final-year manual, and generating new content [29]. Different approaches were chosen for the survey (see figure 1 (Fig. 1)). Supervising physicians were asked about additional support needs regarding teaching in the final year after attending the didactic trainings offered by the faculty. The following main topics were identified through open-ended questions: teaching in clinical practice, legal issues, and using the logbook. The results of a survey on the use of the final-year manual showed that it was rated overall as useful and helpful [30]. In addition, individual and group interviews were conducted with both supervising physicians and students with the question, “What information would you like to have on a website about the final year?” The interviews were evaluated using content analysis [31]. Here, the physicians particularly mentioned information about the assignment of the final-year students on the ward, such as working hours, breakdown of tertials, checklists for interviews, and legal information. The students requested more site-wide information, for example, on legal issues, hygiene and radiation protection regulations, regulations on absences and stays abroad, and support with subsequent applications. In addition, there was a desire for reference materials regarding knowledge specific to the final year, the integration of videos and tutorials, and for faculty-specific information, such as final-year procedures at the respective faculties. For this reason, and because many regulations and forms are faculty-specific, it should also be possible for the individual faculties to include this information in the appropriate places on the website itself.

#### Inventory

The inventory served to identify existing subject areas on the Internet. In addition, a comparison was made of the extent to which the content identified in the needs assessment goes beyond the existing range of information and the website to be developed can offer added value [29]. To this end, German-language platforms on the topic of the final year were searched and analyzed according to the following questions:

- Which groups does the website target?
- What is the website's objective?
- What is the website's content?
- Is the website freely accessible?

It turned out that the existing resources primarily refer to the organization of the final year and are limited to the target group of final-year students. No information has been provided so far for supervising physicians, and large gaps in the area of support for teaching-learning processes were identified.

#### Compilation of the topics

When compiling the topics, the focus was placed on applicability to the final-year situation and the identified needs of the target group [27]. First, important content was identified and coordinated with the medical faculties involved in MERLIN (Freiburg, Heidelberg, Mannheim, Tübingen, Ulm) in planning meetings that also involved students. The content was structured according to the actions required of the final-year students and supervising physicians. Both groups should have access to the basic and practical knowledge they need for practice-related teaching and learning. The content for students was divided into three sections: “vor dem PJ” (before the final year), “im PJ” (during the final year ) and “nach dem PJ” (after the final year). Furthermore, a distinction was made between faculty-specific information, such as local contact persons or registration dates, and site-wide information, such as dates for medical examinations. For the supervising physicians, the focus was on areas such as organization, legal issues and didactic tools. The legal information was compiled by the Competence Center for final-year education in Baden-Württemberg based on current legislation (e.g., § 3 and § 4 ÄApprO 2002, § 276 BGB, § 3 StGB, MuSchuG 2017; StrlSchV 2018) with the involvement of expert representatives (radiation protection officers, hygiene officers, etc.) and consideration of current literature [32], [33], [34].

#### Testing phase

The content and structure were first transferred to presentation software (Microsoft PowerPoint) to simulate the application. This enabled a pre-evaluation to check the navigation and user-friendliness in terms of content and form [35].

**Figure 2:**
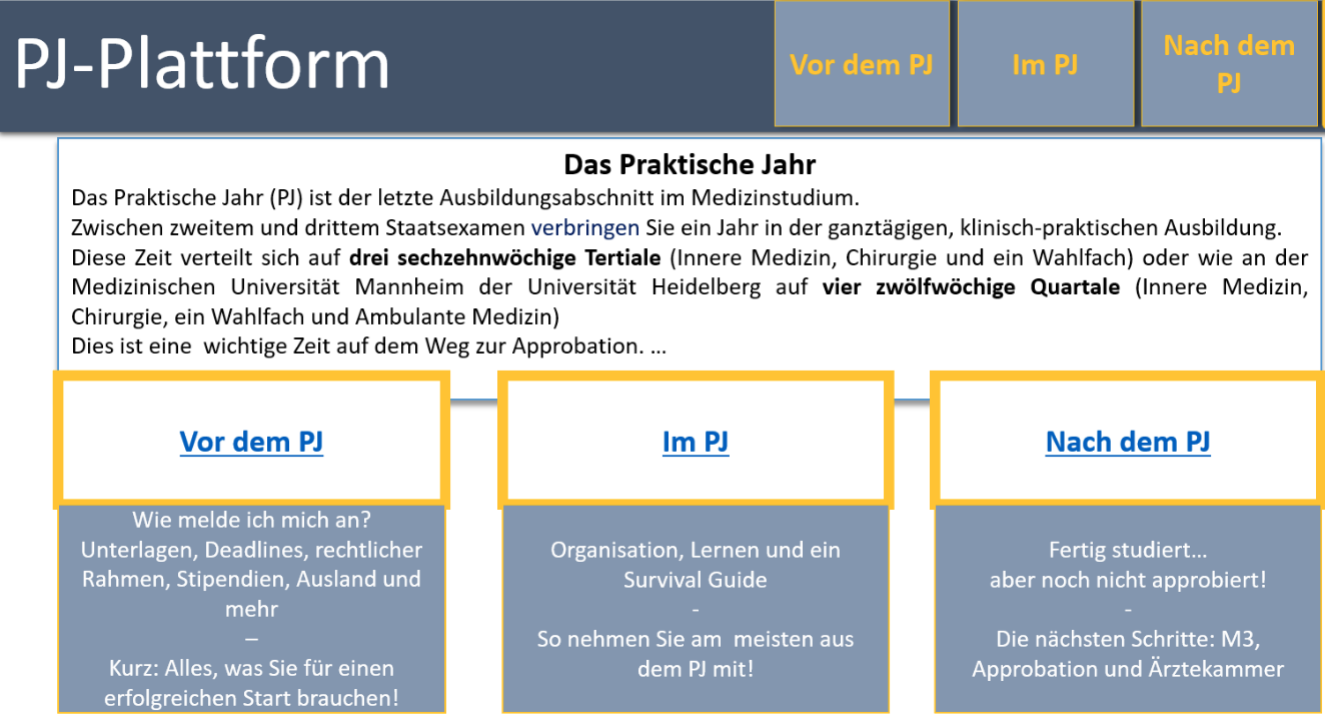
Development of the website (only in German): preliminary structure of the homepage for final-year students

Figure 2 (Fig. 2) shows a first draft for the structure of the homepage for final-year students. The individual modules contain not only organizational information, but also materials that are directly related to the teaching-learning situation.

In the pre-evaluation, two think aloud tests [36], [37] were conducted with two students each, in which they navigated the simulation and answered given and self-formulated questions. The tests lasted approximately 90 minutes each. The click paths and audio track were recorded using a video content management system (Panopto). In addition, students were surveyed in writing regarding the attractiveness, comprehensibility and structure of the simulation, as well as the relevance of the content and the expected benefits [38]. The results of the survey showed that the structure of the site was successful and relevant information was easy to find. Students particularly welcomed the content dedicated to learning in the final year, the “nach dem PJ” (after the final year) section, and the quality and reliability of the information. Criticisms addressed text blocks with a lot of running text and insufficiently prominent inclusion of important documents. Some terms used in the headings were rated as ambiguous and made navigation difficult. Content was also missed, for example, for handling a pregnancy during the final year. Students requested the inclusion of more videos and improved navigation options. A further pretest with supervising physicians was planned, but was not carried out due to a lack of time and personnel resources. Furthermore, this user group had already been clearly included in the needs assessment with regard to content design.

#### Technical realization

The design and implementation took place in cooperation with a professional internet agency. The website was set up as a responsive website using the free content management system (CMS) TYPO3 widely used in the German-speaking world to ensure optimal use on different end devices. Like other CMS systems, TYPO3 CMS consists of a backend, which is used for configuration, development and content maintenance of the website, and a frontend, which represents the website itself. 

The site's page tree was designed and created based on feedback from the pre-evaluation and professional consultation. For the presentation of the content, specific content elements were created as templates based on the predefined content. Only a small number of templates were used to maintain a uniform appearance. Special elements included an interactive map based on the free project OpenStreetMap.org to display possible final-year locations, filtered according to specialization or type of institution, for example. A special content module was also developed to present faculty-specific details to support targeted provision of the most common information (e.g., contact persons, forms, links). 

By means of a specific rights system and the linking of content, the backend was designed in such a way that access to and editing of the faculty-specific portions is possible intuitively and without lengthy training for the responsible persons at the faculties. This low-threshold and simple handling is ensured in that content is entered into a central mask and thus, for example, contact persons at the faculties, who appear several times in the frontend, only have to be entered once in the backend.

#### Design and filling of the website

**Figure 3:**
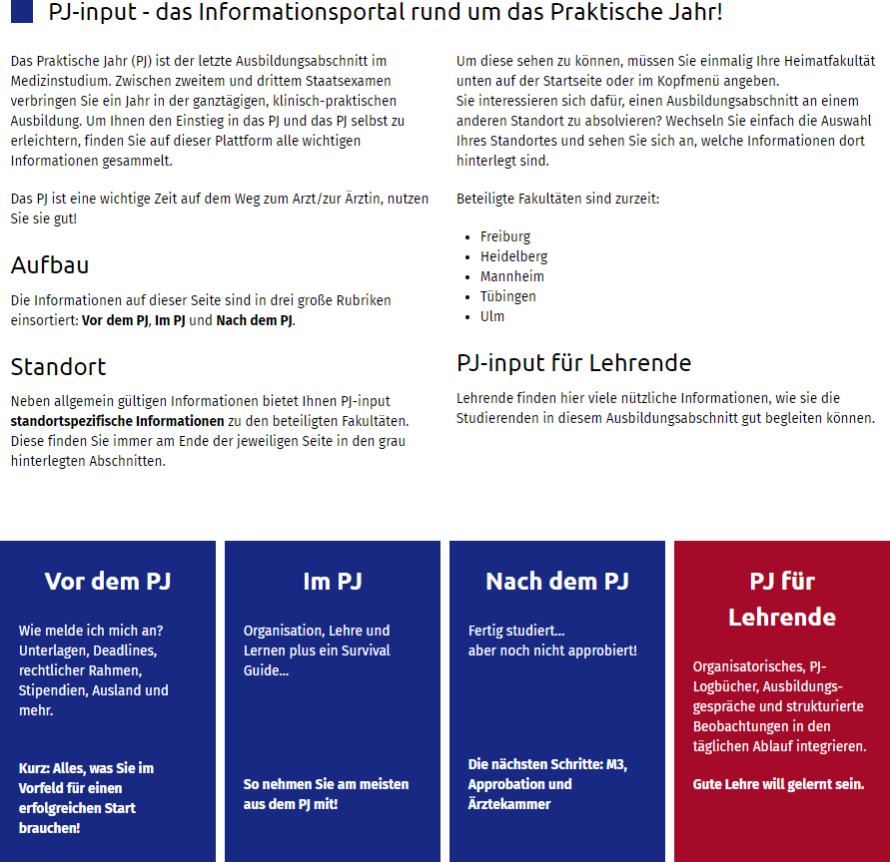
Homepage for *PJ-input *(website only in German)

The design of the texts and the structure of the screen pages were based on the design rules for Internet platforms. For example, great importance was attached to the visual appearance of the screen design, since it has an effect on whether users engage more deeply with a website at all [39]. A logo was developed and a name for the website was assigned in order to achieve a recognition effect, to generate an appropriate URL, and to have a clear designation for legal purposes. The name *PJ-input* was chosen because it is easy to remember and is an acronym composed of the words: Informationen praktisch und transparent (practical and transparent information). This emphasizes the premise of providing information in an application-oriented manner and disclosing commercial or political interests. Appealing visuals were gathered faculty-wide and incorporated into the title bars of each page. Texts for the website were formulated to be as short as possible and easy to read on a screen. Longer texts, such as information on radiation protection, were created as a brochure and included as a printable PDF file. To enable quick and easy orientation and navigation on the website, the sections for students and supervising physicians were specifically color-coded (see figure 3 (Fig. 3)). In addition, faculty-specific information was visually identified. The website was set up so that the desired location could be entered on the homepage. This entry is stored as a cookie for six months on the computers of the users. When used during this period or until a different location is selected, only the information valid for this location is displayed.

#### Staff training

Training was given to the responsible employees at the five participating faculties in Baden-Württemberg so that they can integrate content and media on the website and update and maintain it independently. They should be able to enter content on the faculty-specific pages, such as contact persons, registration deadlines and the respective locations, as well as to update it on an ongoing basis. Access to the backend is also possible via a special entry page with additional support materials.

#### Validation and launch

All content, links, and incorporated materials were reviewed formally and with regard to their relevance, coherence and up-to-dateness by members of the project team and revised as necessary. The final version was released by the responsible person at the Competence Center for final-year education in Baden-Württemberg. The sections for students were released on March 1, 2020, those for supervising physicians on May 2, 2020.

#### Distribution 

The website was advertised through various channels. Final-year students and doctors-in-training were targeted via emails and flyers at the faculties in Baden-Württemberg. Articles in hospital journals and newsletters also contributed to nationwide dissemination. The new logo was integrated into the email signature of the project staff. Due to the pandemic, dissemination at congresses and network meetings could not be implemented as planned.

#### Evaluation

An evaluation tool embedded in the homepage enabled targeted feedback on *PJ-input* during the first year (from March 15, 2020, to March 14, 2021) based on the following open-ended questions:

1. How have you informed yourself about the final year so far?
2. How did you learn about the final-year information platform?
3. What reasons or motivation did you have for visiting the final-year information platform?
4. How could the final-year information platform be improved in your opinion?
5. What content should be changed, corrected or supplemented in your view?
6. Do you have any other comments?

The tool was developed in close cooperation with the Quality Management team at the Mannheim Medical Faculty. 

Furthermore, the usage behavior is continuously recorded via the web applications Webalizer and also with Matomo since July 17, 2021. In each case, the IP addresses are anonymized so that, in compliance with the Data Protection Regulation, it is impossible to draw conclusions about the actual connection.

## Results of the evaluation and usage analysis

During the study period, 21 persons participated in the evaluation integrated on the homepage (final-year students (n=17), supervising physicians (n=3), without indication (n=1)). The low response rate is partly due to the fact that, for data protection reasons, several steps had to be taken in order to participate in the evaluation. The evaluation showed that the students had so far mainly obtained information about the final year from fellow students (n=7). Furthermore, the Internet (n=6), Moodle (n=4), official contacts at the faculty (n=4) and friends (n=2) were mentioned. All participants indicated that they had been made aware of *PJ-input* through official channels, such as email, information sessions or Moodle. When asked about motivation, interest and curiosity (n=4) were mentioned most frequently, but also reasons such as to expand knowledge, to bring oneself up to date, to leave something lasting for future students. One person emphasized that visiting the site was worthwhile and that they wished they had learned about the site before the final year. Suggestions for improvement were mainly related to stronger promotion of the site. Furthermore, the integration of more videos was mentioned as well as the wish that the teaching hospitals should also have a presence. No points of criticism related to the website were mentioned. Inspired by these suggestions, further measures were taken to promote the site (for example, calling on partner faculties to send information emails to students and supervising physicians and to link the site on their learning platforms). Videos on radiation safety and hygiene were also created and included. Since a link to the teaching hospitals’ homepages has already been included in the overview maps of the deployment sites, it is the hospitals’ responsibility to design them accordingly. 

The evaluation of the referrer shows that users mainly access the website via the following three channels: search engines, the homepage of the medical faculties, and their Moodle learning platforms. 

**Figure 4:**
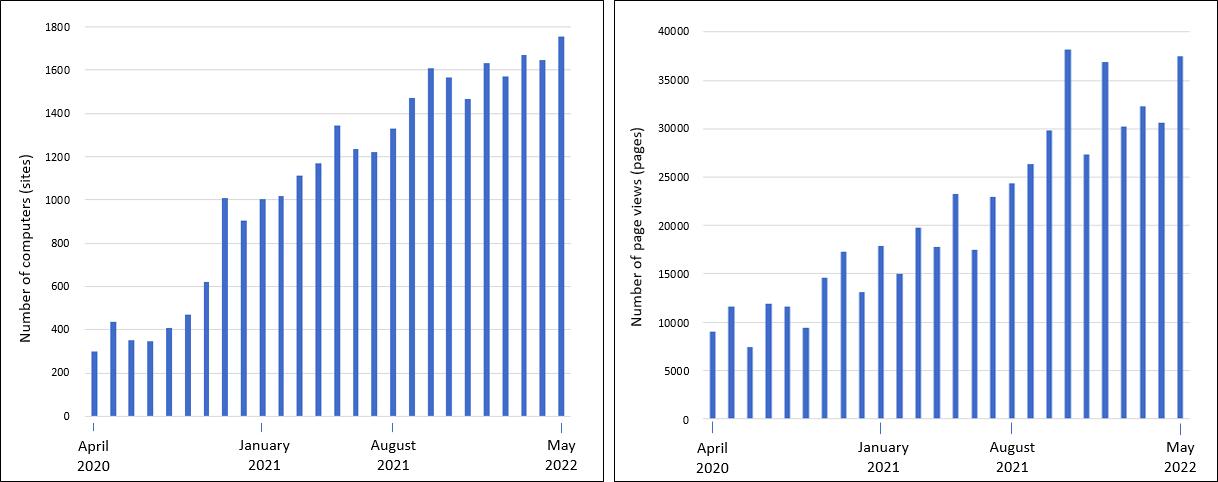
Webalizer statistics on the number of computers and page views per month

Analysis of the Webalizer statistics shows a continuous increase in the number of computers (sites) and page views (pages) (see figure 4 (Fig. 4)).

The number of computers indicates how many IP addresses that directed requests to the server could be identified. This does not mean the exact number of real users, but the number of computers represents the closest determinable number [https://www.lf.net/support/techinfo/webserver/webalizer.php]. Therefore, based on the statistics, it can be concluded that about 1,700 people are currently accessing *PJ-input* per month. The evaluation of the web analytics shows that mainly information on organizational issues was searched for and that the focus was on site-wide pages in the student section. Examples of the most frequently entered search terms and hits entail the expense allowances in the final year and the third state examination. 

**Figure 5:**
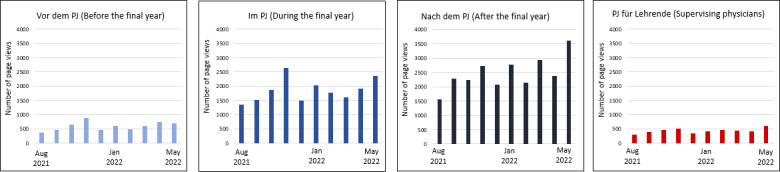
Matomo statistics on the average number of page views per month per section for the periods August 2021 to May 2022

The section-specific evaluation via Matomo shows that mainly the sections “im PJ” (during the final year) and “nach dem PJ” (after the final year) are used and that the sections “vor dem PJ” (before the final year) and “PJ für Lehrende” (final-year information for supervising physicians) are visited less frequently (see figure 5 (Fig. 5)).

An evaluation of the website’s distribution shows that it is mainly used within Germany in Baden-Württemberg, but also in other German states in the southwest (Rhineland-Palatinate and Saarland) and north (Schleswig-Holstein, Brandenburg, Berlin, Mecklenburg-Western Pomerania). No use is recorded for the other German states. 

Further maintenance of *PJ-input* is the responsibility of the Competence Center for final-year education located in Mannheim. Here, the information and links provided are regularly checked to ensure that they are up to date, and suggestions for improvement are reviewed and implemented if necessary. The involvement of other faculties to provide faculty-specific information and open-source materials is expressly welcomed.

## Discussion

The elaborate and professional development of *PJ-input* was federally funded in order to enjoy national benefit with regard to competency-oriented training in medical studies. The information platform offers secured information in a non-commercial context and is focused on the needs of the target groups. The integration of a variety of expertise (content-related, professional and technical) as well as the coordination of the content across several faculties reduced the development costs to create materials, such as the videos posted on the website [40]. In this way, it was also possible to achieve generalizability of the content, so that *PJ-input* can be used throughout Germany. The site-wide and low-threshold provision of didactic materials and information addresses current developments in medical studies and represents a unique selling point for this website. The heavy use of the “nach dem PJ” (after the final year) section shows that students are actively looking for information on the transition from study to career.

A needs assessment was carried out in order to tailor the website to the needs of the students. Here, there is a fundamental risk that needs will be interpreted into it [41]. The same applies to the think aloud method [35]. As the evaluation of the web analytics shows, the website has been well received so far, so it can be assumed that the needs of the users were anticipated well. The timing of the website launch coincided with the beginning of the pandemic. Here, it proved advantageous that the website is easy to maintain, so that changes required at short notice could be responded to immediately and corresponding faculty-specific details could also be communicated.

Overall, however, development and implementation required a great deal of staff time, leaving few resources for distribution or for involving other faculties from other states, for example, in posting faculty-specific information. And the pandemic situation at the start of *PJ-input* prevented nationwide distribution as intended by the project. As a result, the website is not (yet) accessed in some federal states. Convincing was also necessary among the project partners in Baden-Württemberg, since information about the final year is usually provided on the respective homepages or learning platforms of the faculties and thus the benefit of another website was not recognized initially. Therefore, the possibilities of *PJ-input* in the support and competency-based orientation of teaching must be made more visible. Overall, greater and more widespread use is desirable. By involving more faculties and their expertise, the site could be enriched, for example, with more teaching videos, links and informational materials, so that elaborately developed materials can be used across faculties. It is expected that the introduction of the new licensing regulations will increase the need for information among faculty and students. Especially with the introduction of competency-based teaching in the final year, the *PJ-input* could help to fill information gaps and simplify and standardize the implementation of EPA. At the same time, there is the challenge of keeping *PJ-input* constantly up to date and adapting it to changing conditions. 

The increasing number of visitors and the positive feedback show that the project goal of providing information to support learning in the final year for all supervising physicians and students has been achieved. The high usage rate by students indicates that there is a corresponding need for digitally accessible support. The site must be further disseminated among supervising physicians so that it can make a lasting contribution to their professionalization.

## Funding

The project was funded by the German Federal Ministry of Education and Research (BMBF) under project number 01PL17011C and 01PL1701D.

## Acknowledgement

We would like to thank our partners for their cooperation, all of the medical faculties who supplied us with informational materials and constructive criticism, the students who participated in the pre-evaluation, and the project collaborators Mara Geißinger, Nicolas Krapp and Mareike Pieper, who contributed significantly to its successful implementation.

## Competing interests

The authors declare that they have no competing interests.
